# Higher risk of traumatic intracranial hemorrhage with antiplatelet therapy compared to oral anticoagulation—a single-center experience

**DOI:** 10.1007/s00068-024-02493-z

**Published:** 2024-03-21

**Authors:** Emily Niklasson, Elin Svensson, Lars André, Christian Areskoug, Jakob Lundager Forberg, Tomas Vedin

**Affiliations:** 1 Clinical Sciences, Malmö, Clinical Research Centre, CRC, Lund University, Plan 11, Jan Waldenströms Gata 35, Malmö, Sweden; 2 Clinical Sciences, Helsingborg, Lund University, Svartbrödragränden 3-5, 251 87 Helsingborg, Sweden

**Keywords:** MESH, Brain injuries, Traumatic intracranial hemorrhages, Traumatic anticoagulants tomography, X-ray computed

## Abstract

**Purpose:**

Traumatic brain injury is the main reason for the emergency department visit of up to 3% of the patients and a major worldwide cause for morbidity and mortality. Current emergency management guidelines recommend close attention to patients taking oral anticoagulation but not patients on antiplatelet therapy. Recent studies have begun to challenge this. The aim of this study was to determine the impact of antiplatelet therapy and oral anticoagulation on traumatic intracranial hemorrhage.

**Methods:**

Medical records of adult patients triaged with “head injury” as the main reason for emergency care were retrospectively reviewed from January 1, 2017, to December 31, 2017, and January 1, 2020, to December 31, 2021. Patients ≥ 18 years with head trauma were included. Odds ratio was calculated, and multiple logistic regression was performed.

**Results:**

A total of 4850 patients with a median age of 70 years were included. Traumatic intracranial hemorrhage was found in 6.2% of the patients. The risk ratio for traumatic intracranial hemorrhage in patients on antiplatelet therapy was 2.25 (*p* < 0.001, 95% confidence interval 1.73–2.94) and 1.38 (*p* = 0.002, 95% confidence interval 1.05–1.84) in patients on oral anticoagulation compared to patients without mediations that affect coagulation. In binary multiple regression, antiplatelet therapy was associated with intracranial hemorrhage, but oral anticoagulation was not.

**Conclusion:**

This study shows that antiplatelet therapy is associated with a higher risk of traumatic intracranial hemorrhage compared to oral anticoagulation. Antiplatelet therapy should be given equal or greater consideration in the guidelines compared to anticoagulation therapy. Further studies on antiplatelet subtypes within the context of head trauma are recommended to improve the guidelines’ diagnostic accuracy.

## Introduction

Traumatic brain injury (TBI) is a common cause for seeking emergency care, and up to 3% of the emergency department (ED) patients are cared for because of it [1]. It is also a cause for major morbidity and mortality in both adults and children [2, 3]. However, early signs and symptoms do not always correlate with injury severity, making emergency management of TBI challenging to ED physicians [4, 5]. Accurate diagnosis can be achieved with computerized tomography (CT) of the head. However, this exposes the patient to ionizing and potentially harmful radiation as well as leading to increased costs and prolonging management time [6–9]. Only up to 10% of ED TBI patients are diagnosed with a traumatic intracranial hemorrhage (TICH) [10].

Epidemiology of TBI has changed over the past 30 years. The patients are older, have more comorbidities, and take more medications affecting coagulation, and the primary trauma mechanism has shifted from motor vehicle accidents to falls [11]. For instance, while earlier reports indicated the lowest prevalence of TBI in the 50–60 years age group, recent studies have presented converse results [1, 10].

Use of antiplatelet therapy (APT) and anticoagulation therapy (ACT) is widespread in elderly patients. However, the prescription of APT and vitamin K antagonists (VKA) is decreasing, and the prescription of direct oral anticoagulants (DOAC) is increasing. In the year 2022, the prescription rate of DOAC in Sweden had doubled since the year 2018, but prescription of APT was still 45% higher than that of DOAC and VKA combined [12, 13].

APT has until recently been regarded to confer a smaller risk of TICH than ACT [14–16]. Nevertheless, a few publications have reported converse results with more TICHS found in patients taking APT than in patients taking ACT [1, 17–19]. TICH rates might be lower in patients medicated with DOACs than in patients taking VKAs. Rates have been reported between 10–40% in patients on APT and 5–20% in patients medicated with ACT [1, 20–25]. More recent studies have found that up to 10% of head trauma patients > 65 years may not show any symptoms of head injury but are still diagnosed with a TICH, notwithstanding pharmaceutical treatment that affects coagulation [26, 27]. Because of this, Schindler et al. (2023) have argued that a head CT is mandatory in these patients [27].

This increased prevalence of TICH in patients on ACT compared to those without treatment affecting coagulation is considered in current guidelines for initial management of TBI patients. These guidelines, such as the Canadian CT Head Rule (CCHR), the New Orleans Criteria (NOC), the Eastern Association for the Surgery of Trauma (EAST), and Neurotrauma Committee Guidelines (SNC), consistently recommend a head CT in patients receiving ACT [14, 16, 28, 29]. In an update of the National Institute for Health and Care Excellence (NICE) guidelines from 2023, a head CT is no longer mandatory but still considered in these patients, reflecting that a shift might be underway [30]. However, the NICE guidelines do not mandate a head CT for all patients < 65 years [30].

To mitigate the risk of delayed intracranial hemorrhage, the SNC guideline recommends hospitalization of patients on ACT even if initial head CT rules out TICH and EAST makes this recommendation in patients on ACT when they have supratherapeutic international normalized ratio [16, 29]. Conversely, with the exception of SNC, current guidelines do not recommend different management to patients on APT than patients without medication affecting coagulation [16]. In general, the sensitivity of current guidelines is high, but the specificity is low (12–58%) [31, 32].

The shift in trauma mechanism and older age might also impact the rate of TICHs. Trauma energy is only considered by the CCHR. Fakhry et al. (2021) studied a trauma registry with 33,000 geriatric patients with ground-level falls. Their conclusion was that APT and DOAC were not linked to an increased risk of TICH but dual APT (aspirin-clopidogrel) was [33]. If confirmed by additional studies, these findings suggest that guidelines may warrant revision, with antiplatelet therapy (APT) potentially meriting equal or more emphasis in diagnostic workup of head trauma patients compared to anticoagulation therapy (ACT).

The reason for performing the present study was that the epidemiological and pharmaceutical changes may have altered which TBI patients that are at risk of sustaining TICHs.

The aim of this study was to determine the impact of antiplatelet therapy and oral anticoagulation on traumatic intracranial hemorrhage.

## Materials and methods

Data was retrospectively gathered by reviewing the medical records of patients with isolated head trauma at the Helsingborg General Hospital ED. The hospital recommends the SNC guideline for emergency management of head trauma patients [16]. The catchment area of 275,000 people generates about 70,000 ED visits per year. The neurosurgical ward is situated 40 km away at the university hospital in Lund. Data collection was performed from January 1, 2017, to December 31, 2017, and from January 1, 2020, to December 31, 2021. The database was collected as part of a more comprehensive research project, and the gap years 2018 and 2019 were not pertinent to the present study. Patients screened for participation in this study were identified in the digital ED ledger if they had “head trauma” as the presenting complaint.

The inclusion criteria were as follows:

- Presenting complaint “head trauma” (triaged by emergency department nurse)
- Age ≥ 18 years

Exclusion criteria were as follows:

- Planned return-visits/checkups
- Identical, classified, or empty medical records
- Management by nurse without physician’s participation
- Minor/other visits/no head trauma found when reviewing medical records (e.g., minor trauma with skin laceration in need of sutures without trauma to the neurocranium where the patient was not assessed by the managing physician because of a trauma to the brain)

The included patients may have had additional injuries. However, if their initial triage classification was “multitrauma,” they were never screened for study participation.

The following parameters were collected and analyzed in the present study:

1. Age (years)
2. Gender (female/male)
3. Charlson Comorbidity Index
4. CT of the head performed (yes/no)
5. Outcome of CT of the head (intracranial hemorrhage/no intracranial hemorrhage)
6. Admission to hospital (yes/no)
7. Admission to intensive care unit or neurointensive care unit (yes/no)
8. Neurosurgical intervention or intubation (yes/no)
9. Level of consciousness using Glasgow Coma Scale (GCS)
10. ACT (no/VKA/DOAC/low-molecular weight heparin (LMWH))
11. APT (no/acetylsalicylic acid/clopidogrel/ticagrelor/prasugrel/dipyramidol/combinations)
12. Intoxication
13. New focal neurological deficits (yes/no)
14. Nausea (yes/no)
15. Vomiting (yes/no)
16. Amnesia (yes/no)
17. Loss of consciousness (yes/no)
18. Peritraumatic seizure (yes/no)
19. Scalp hematoma (yes/no)
20. Signs of fracture of skull or base of skull (yes/no)

Parameters GCS, intoxication, new focal neurological deficits, nausea, vomiting, amnesia, loss of consciousness, peritraumatic seizures, scalp hematoma, and signs of fracture were obtained from the physical examination performed by the managing physician upon the first evaluation.

Numerous inclusions of the same patient were possible if the patient presented on various occasions with different traumas.

Primary outcome measure was relative risk for traumatic intracranial hemorrhage. Secondary outcome measure was odds ratio for factors associated with traumatic intracranial hemorrhage.

Patients’ comorbidity load was quantified with Charlson Comorbidity Index (CCI). It is validated for prospective and retrospective application [34–37].

Helsingborg Hospital is part of Region Skåne, a geographical region in the south of Sweden where six hospitals with emergency departments share an electronic system for medical records. Records from the emergency visit, the ward (if admitted), laboratory, and radiology reports from the entire Skåne region were surveyed. If medications and comorbidities were not stated in the ED reports, records 1 year before the ED visit from the whole region were reviewed. When other data was missing in physician’s ED notes, other staffs’ (e.g., paramedics or nurses) notes were reviewed. Prospective review of the notes 6 months after the ED visit was performed to ensure that no TICHS were missed. Total sum of follow-up visits each patient had was not noted.

A total of seven reviewers collected data. Guidelines for retrospective review of medical records by Vassar and Holzman were followed to reduce information bias [38]. This entailed producing and adhering to an extensive pro forma disambiguating data interpretation and coding. It also stated with well-defined criteria for inclusion and exclusion. To investigate data validity, we previously published an analysis of Cohen’s kappa coefficient was completed in 100 randomized records that were scrutinized by two researchers [21]. All parameters except new neurological deficits and LMWH treatment showed good/very good agreement.

### Data definitions and missing data

Intracranial hemorrhage found by head CT scan at index ED visit was defined as TICH. Not finding any signs of intracranial hemorrhage on index CT or treating physician not ordering a CT was defined as absence of TICH. Intervention was defined as surgical intervention, endotracheal intubation, or intensive care because of the head trauma. Mortality because of TBI was defined as any death that was caused by the head injury that occurred during the hospital stay. One or several pharmaceuticals affecting thrombocyte function and no concurrent ACT was defined as APT. Any pharmaceutical affecting coagulation factors and no concurrent APT were defined as ACT. ACT was subsequently divided into a VKA cohort and a DOAC cohort. LMWH was defined as any treatment with LMWH and no concurrent APT and/or ACT. Any combinations of APT and ACT were denoted “combination of antiplatelet and anticoagulation therapy,” and these patients were not included in any analysis of patients on ACT or APT.

Loss of consciousness was interpreted as “yes” if witnessed or if patient assumed that it has occurred. Vomiting was interpreted as “yes” if any episode of vomiting had occurred until the physician examined the patient or if it was stated in the medical records that the patient started vomiting after the physician had seen the patient. Amnesia was interpreted as “yes” if the patient had difficulties remembering the time leading up to the trauma and/or the time after the trauma.

Missing data was coded as missing but analyzed pragmatically by construing it as the lack of pathology (e.g., no mention of amnesia was analyzed as “no amnesia”). This was done except for level of consciousness which was analyzed as missing when missing. This way of managing missing data was established after a thorough group discussion concluding that, in our experience, ED doctors write rational records, reporting positive findings without negating negative findings. The parameters relevant to the present study are regularly evaluated when managing TBI patients, and this was therefore considered an acceptable risk of information bias.

For international eligibility, level of consciousness was reported as Glasgow Coma Scale (GCS) level. Swedish standard is to report according to Reaction Level Scale (RLS). GCS level was obtained by conversion between RLS and GCS. Previous research has shown good agreement between RLS1-2 and GCS15-14 but worse agreement between RLS3-8 and GCS 13–3 [39]. Because of this, level of consciousness was reported as GCS ≥ 14 or < 14.

### Statistical analysis

Data analysis was performed with SPSS version 27 for Mac. Histograms and Shapiro–Wilk’s test were used to delineate data distribution, and non-parametric parameters were presented with median, 25th and 75th percentiles (Q1, Q3).

The risk ratio (RR) for TICH with a 95% confidence interval (CI) was calculated for the APT and ACT cohorts compared to patients without treatment medication affecting coagulation. Percentage proportions of TICH in different drug classes were analyzed with 95% CI. The dispersion of TICH in these cohorts was probability tested with *χ*^2^ test. *p* < 0.05 was regarded as statistically significant with the exception in first step of the binary logistic regression.

A binary logistic regression was executed to evaluate if outcomes of risk ratio analysis and *χ*^2^ testing were obtained because of confounding, or a real association obtained in a multivariate exploration.

Characteristics generally related to TICH, CCI, and DOAC/VKA/APT were used as independent variables. CCI cutoff was pragmatically obtained by analysis of each step in the scale with univariate binary logistic regression and choosing the one with highest odds ratio (OR) and lowest CI. Age was divided into groups of 10-year intervals, and optimal cutoff was obtained in the same way as for CCI. The purpose of this was to find the cutoff with the most effect on TICH. The binary logistic regression was executed as a three-step process with the univariate logistic regression with TICH as dependent variable in the first step. The second step (denoted “multiple regression 1” in regression tables) entailed that all independent variables with *p* < 0.4 from the first step were analyzed in a multiple binary logistic regression. The last step (denoted “multiple regression 2” in regression tables) was analyzed as a multiple binary logistic regression with all variables that achieved *p* < 0.05 in the second analysis. *p*-value and OR with 95% CI were stated for each of the three steps. The determination coefficient was stated as Nagelkerke *R*^2^.

## Results

**Fig. 1:**
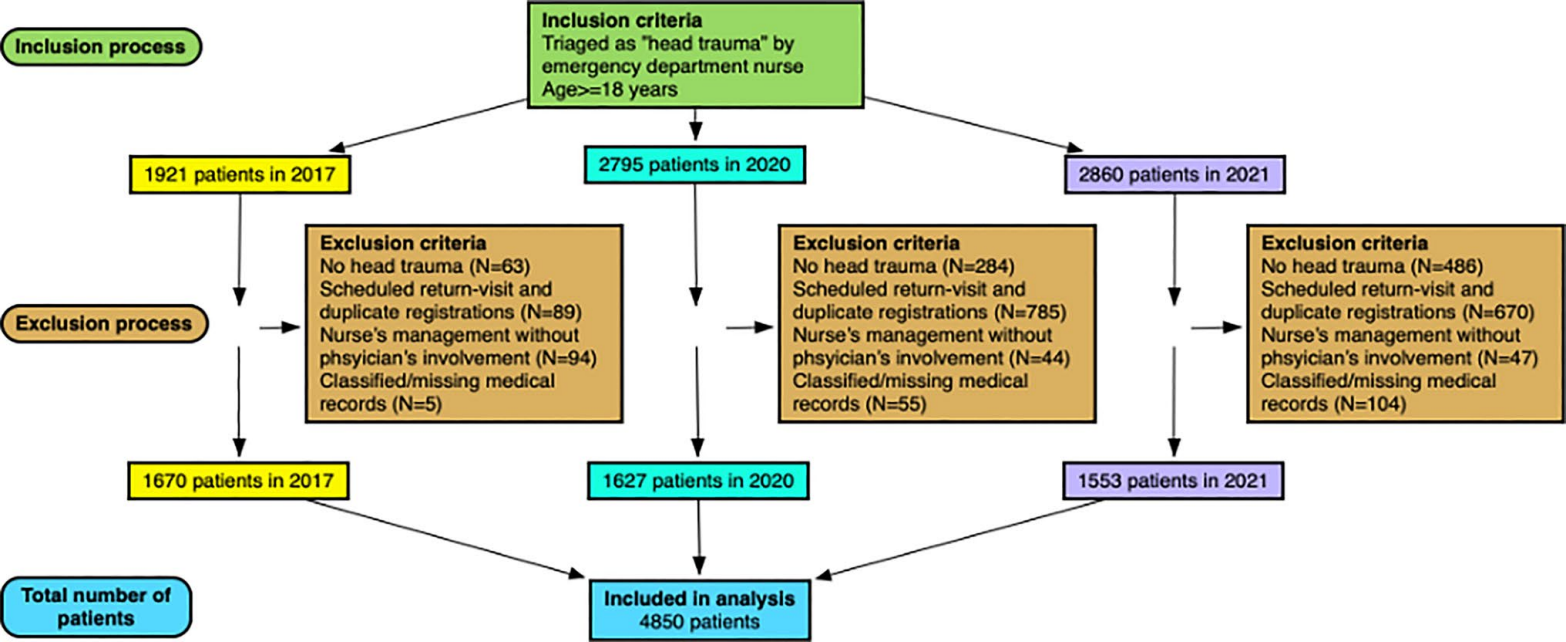
Inclusion process

A total of 4850 head trauma patients were included in the study. See Fig. 1 for inclusion process.

Figure 1 shows inclusion process of the entire study with 1670 included patients from 2017, 1627 from 2020, and 1553 from 2021.

A total of 302/4850 (6.2%, 95% CI 5.6–6.9%) patients were diagnosed with TICH, and 140 of these were on APT or ACT. TICH was found in 153 patients (4.9%, 95% CI 4.2–5.7%) patients not on medication affecting coagulation, in 66 (6.8%, 95% CI 5.3–8.5%) patients receiving ACT, and in 74 (11.0%, 95% CI 8.7–13.6%) patients on APT. In the LMWH-treatment subgroup, 4/30 patients (13.3%, 95% CI 4.8–31.0%) had TICH, and in the subgroup combining ACT and APT, 4/28 patients (12.5%, 95% CI 3.5–29.0%) had TICH.

Patients with TICH had a median age of 77 (64, 86) years. See Table 1 for general data on the population and Table 2 for general data and comparison of patient characteristics in patients with TICH.

**Table 1:** Characteristics of all patients

Variable name	All patients (*n* = 4850)	Patients without therapy affecting coagulation (*n* = 3136)	Anticoagulation therapy (*n* = 975)	Antiplatelet therapy (*n* = 673)
Age (median (quartile 1, quartile 3)) years	70 (46, 83)	55 (33, 73)	84 (77, 89)	80 (73, 87)
Gender				
Male *n* (% of entire population)	2466 (50.8%)	1640 (52.3%)	457 (46.9%)	334 (49.6%)
Female *n* (% of entire population)	2384 (49.2%)	1496 (47.7%)	518 (53.1%)	339 (50.4%)
Glasgow Coma Scale (GCS)*				
GCS 14–15 *n* (% of entire population)	4730 (99.0%)	3047 (99%)	959 (99.5%)	659 (98.8%)
GCS < 14 *n* (% of entire population)	46 (1.0%)	32 (1.0%)	5 (0.5%)	8 (1.2%)
Patient history				
Vomiting *n* (% of entire population)	340 (7.0%)	278 (8.9%)	33 (3.4%)	28 (4.2%)
Loss of consciousness *n* (% of entire population)	1430 (29.5%)	1006 (32.1%)	212 (21.8%)	195 (29%)
Amnesia *n* (% of entire population)	1194 (24.6%)	847 (27%)	183 (18.8%)	150 (22.3%)
Charlson Comorbidity Index				
< 7 *n* (% of entire population)	4821 (99.4%)	3128 (99.7%)	965 (99.0%)	665 (98.8%)
≥ 7 *n* (% of entire population)	29 (0.6%)	8 (0.3%)	10 (1.0%)	8 (1.2%)
Clinical findings, outcomes, and interventions				
New focal neurological deficits *n* (% of entire population)	270 (5.6%)	183 (5.8%)	41 (4.2%)	44 (6.5%)
Peritraumatic seizures *n* (% of entire population)	77 (1.6%)	53 (1.7%	10 (1.0%)	12 (1.8%)
Traumatic intracranial hemorrhage *n* (% of entire population)	302 (6.2%)	153 (4.9%)	66 (6.8%)	74 (11%)
Head computerized tomography performed *n* (% of entire population)	3360 (69.3%)	1763 (56.2%)	926 (95%)	608 (90.3%)
Admission to hospital *n* (% of entire population)	1556 (32.3%)	594 (18.9%)	664 (68.1%)	263 (39.1%)
Intervention*** during index visit *n* (% of entire population)	28 (0.6%)	17 (0.5%)	3 (0.3%)	4 (0.6%)
Death due to head injury *n* (% of entire population)	27 (0.6%)	13 (0.4%)	8 (0.8%)	5 (0.7%)

*Variable Glasgow Coma Scale has 74 missing cases

**Cutoff obtained by choosing the scale step of Charlson Comorbidity Index that had the highest odds ratio and narrowest confidence interval in regression of Charlson Comorbidity Index and traumatic intracranial hemorrhage

***Neurosurgical operation, intubation, or care at intensive care unit because of head injury. The 30 cases with low-molecular weight heparin treatment and the 32 cases with combinations of anticoagulation and antiplatelet therapy were not included in table

**Table 2:** Comparison of all patients with traumatic intracranial hemorrhage

Variable name and *p*-value	Patients without therapy affecting coagulation (*n* = 153)	Anticoagulation therapy (*n* = 66)	Antiplatelet therapy (*n* = 74)
Age (median (quartile 1, quartile 3)) years	68 (51, 80)	84 (77, 89)	86 (78, 90)
*p*-value (Mann–Whitney *U* test)	< 0.001 and < 0.001^1^	0.029^2^	
Gender			
Male *n* (% of entire population)	75 (49.0%)	38 (57.6%)	43 (58.1%)
Female *n* (% of entire population)	78 (51.0%)	28 (42.4%)	31 (41.9%)
*p*-value (*χ*^2^-test)	0.13	0.43	0.34
Glasgow Coma Scale (GCS)			
GCS 14–15 *n* (% of entire population)	137 (91.3%)	963 (95.5%)	68 (94.4%)
GCS < 14 *n* (% of entire population)	13 (8.7%)	6 (9.1%)	4 (5.6%)
*p*-value (*χ*^2^ test)	0.28	0.36	0.56
Patient history			
Vomiting *n* (% of entire population)	29 (19%)	6 (9.1%)	3 (4.1%)
*p*-value (*χ*^2^ test)	**0.02**	0.3	**0.009**
Loss of consciousness *n* (% of entire population)	77 (50.3%)	23 (34.8%)	32 (43.2%)
*p*-value (*χ*^2^ test)	0.06	0.06	0.72
Amnesia *n* (% of entire population)	63 (41.2%)	23 (34.8%)	23 (31.1%)
*p*-value (*χ*^2^ test)	0.17	0.63	0.2
Charlson Comorbidity Index			
< 8 *n* (% of entire population)	1 (0.7%)	2 (3%)	0 (0%)
≥ 8 *n* (% of entire population)	152 (99.3%)	64 (97%)	74 (100%)
*p*-value (*χ*^2^ test)	0.55	0.06	0.32
Clinical findings, outcomes, and interventions			
New focal neurological deficits *n* (% of entire population)	22 (14.4%)	9 (13.6%)	10 (13.5%)
*p*-value (*χ*^2^ test)	0.81	0.3	0.81
Peritraumatic seizures *n* (% of entire population)	8 (5.2%)	3 (4.5%)	6 (8.1%)
*p*-value (*χ*^2^ test)	0.59	0.59	0.4
Admission to hospital *n* (% of entire population)	139 (90.8%)	61 (92.4%)	66 (89.2%)
*p*-value (*χ*^2^ test)	0.94	0.59	0.6
Intervention^3^ during index visit *n* (% of entire population)	11 (7.2%)	3 (4.5%)	4 (5.4%)
*p*-value (*χ*^2^ test)	0.52	0.51	0.72
Death due to head injury *n* (% of entire population)	11 (7.2%)	7 (10.6%)	4 (5.4%)
*p*-value (*χ*^2^ test)	0.78	0.3	0.41

Table shows that there are no statistically significant differences in patient characteristics apart from age and vomiting

^1^Compared to both therapy groups

^2^Comparing anticoagulation therapy group with antiplatelet therapy group to each other

3Treatment at intensive care unit, intubation, or neurosurgical operation because of head injury

The most common trauma mechanism was fall from ground level (2944 patients, 60.7%), followed by abuse (246 patients, 5.1%), bike accidents (244 patients, 5.0%), fall from < 1 m (157 patients, 3.2%), fall from 1–3 m (157 patients, 3.2%), hit by flying object (155 patients, 3.2%), motor vehicle accident (134 patients, 2.8%), running into stationary object (119 patients, 2.5%), sports injury (99 patients, 2.0%), pedestrian hit by vehicle (22 patients, 0.5%), and fall from > 3 m (19 patients, 0.4%). A total of 554 patients (11.4%) had an unclear trauma mechanism.

APT was found in 673 patients (13.8%), with acetylsalicylic acid (ASA) being the predominant APT drug class with 540/673 (80.2%) patients. ACT was identified in 974 (20.1%) of all patients. DOAC was the most common ACT found in 688 (70.6%) patients, while VKAs were found in 286 (29.3%) patients. See Table 3 for distribution of TICHs and pharmaceutical treatment affecting coagulation.

**Table 3:** Drugs affecting coagulation and rate of traumatic intracranial hemorrhage

Type of medication	No intracranial hemorrhage (% of each drug class)	Intracranial hemorrhage (% of each drug class)
Antiplatelet therapy	**599 (89.0%)**	**74 (11%)**
Acetylsalicylic acid	480 (88.9%)	60 (11.1%)
Clopidogrel	88 (88.9%)	11 (11.1%)
Ticagrelor	2 (100%)	0 (0.0%)
Cilostazol	24 (96.0%)	1 (4.0%)
Dual antiplatelet therapy	5 (71.4%)	2 (28.6%)
Anticoagulation therapy	**909 (93.2%)**	**66 (6.8%**
Warfarin	286 (95.7%)	13 (4.3%)
Apixaban	479 (92.3%)	40 (7.7%)
Dabigatran etexilate	35 (92.1%)	3 (7.9%)
Rivaroxaban	100 (90.9%	10 (9.1%)
Edoxaban	9 (100%)	0 (0.0%)
Low-molecular weight heparin therapy	**26 (86.7%)**	**4 (13.3%)**
Tinzaparin	22 (84.6%)	4 (15.4%)
Enoxaparin	4 (100%)	0 (0.0%)
Combination of antiplatelet and anticoagulation therapy		
*	28 (87.5%)	4 (12.5%)
Combination of low-molecular weight heparin and antiplatelet or anticoagulation therapy		
**	3 (100%)	1 (0.0%)
No antiplatelet or anticoagulation therapy		
-	2983 (95.1%)	153 (4.9%)

Table showing distribution of patients on different drugs within the drug classes “antiplatelets,” “anticoagulation,” and low-molecular weight heparin and combinations thereof and in patients without drug therapy affecting coagulation

*Combinations of acetylsalicylic acid, clopidogrel, ticagrelor, or prasugrel and warfarin, apixaban, dabigatran, or edoxaban

**Either single therapy of tinzaparin or enoxaparin and any combination of acetylsalicylic acid, clopidogrel, ticagrelor, or prasugrel and warfarin, apixaban, dabigatran, or edoxaban

The RR for TICH compared to those without medication affecting coagulation was 2.25 (*p* < 0.0001, 95% CI 1.73–2.94) in APT patients and 1.38 (*p* = 0.02, 95% CI 1.05–1.84) in ACT patients

The rate of TICH in patients on APT was significantly higher compared to patients without coagulation-affecting therapy (*χ*^2^ test *p* < 0.0001). Similarly, the rate of TICH in patients on ACT was significantly higher compared to that in patients without coagulation-affecting therapy (*χ*^2^ test *p* < 0.02)

See Tables 4 and 5 for logistic regression analysis of factors commonly associated with TICH

**Table 4:** Regression analysis of factors commonly associated with traumatic intracranial hemorrhage with anticoagulation therapy as independent variable

Variable	Univariate regression		Multiple regression 1		Multiple regression 2	
	*p*	OR^1^ (95% CI^2^)	*p*	OR^1^ (95% CI^2^)	*p*	OR^1^ (95% CI^2^)
Gender	0.38	1.11 (0.88–1.40)	0.38	1.12 (0.87–1.43)	-	
Age > 45	< 0.001	3.28 (2.22–4.84)	< 0.001	3.59 (2.36–5.46)	< 0.001	3.54 (2.33–5.38)
Charlson Comorbidity Index > 7	< 0.001	7.62 (2.28–25.44)	0.002	6.98 (2.03–24.01)	0.002	7.35 (2.14–25.20)
Anticoagulation therapy	0.43	1.12 (0.84–1.49)	-	-	-	
Antiplatelet therapy	< 0.001	2.14 (1.62–2.82	< 0.001	1.66 (1.23–2.23)	< 0.001	1.66 (1.24–2.24)
Glasgow Coma Scale < 14	< 0.001	13.56 (7.49–24.52)	< 0.001	11.14 (5.55–22.36)	< 0.001	11.47 (5.72–22.99)
Loss of consciousness	< 0.001	2.06 (1.63–2.61)	0.002	1.55 (1.17–2.05)	0.002	1.56 (1.18–2.06)
Amnesia	< 0.001	1.92 (1.50–2.45)	< 0.001	1.73 (1.30–2.31)	< 0.001	1.75 (1.31–2.33)
Vomiting	< 0.001	2.09 (1.47–2.99)	< 0.001	2.03 (1.36–3.03)	< 0.001	2.01 (1.35–2.99)
Headache	0.68	1.06 (0.81–1.40)	-	-	-	
Seizure	< 0.001	4.82 (2.81–8.29)	0.005	2.44 (1.31–4.56)	0.005	2.44 (1.31–4.55)
Intoxication	0.42	0.82 (0.66–1.19)	-		-	
Scalp hematoma	0.005	1.53 (1.14–2.07)	0.025	1.43 (1.05–1.97)	0.028	1.42 (1.04–1.95)
Signs of open/depressed fracture	0.26	0.66 (0.32–1.36)	0.6	0.82 (0.40–1.71)		
Signs of skull base fracture	< 0.001	3.19 (2.07–4.90)	< 0.001	2.77 (1.76–4.36)	< 0.001	2.77 (1.76–4.37)
New neurological deficits	< 0.001	3.06 (2.15–4.35)	< 0.001	2.09 (1.40–3.15)	< 0.001	2.10 (1.40–3.15)

Table shows regression analysis in three steps. “Univariate regression” is univariate logistic regression of all relevant parameters. Multiple regression 1 is multiple logistic regression with all parameters from univariate regression that had *p* < 0.4. Multiple regression 2 is multiple logistic regression with all parameters from multiple regression 1 that had *p* < 0.05. Analysis shows that anticoagulation therapy is not associated with intracranial hemorrhage, but antiplatelet therapy is. *R*^2^ = 0.13

^1^Odds ratio

^2^Confidence interval

**Table 5:** Regression analysis of factors commonly associated with traumatic intracranial hemorrhage with anticoagulation therapy separated into two categories (warfarin and other direct oral anticoagulants)

Variable	Univariate regression		Multiple regression 1		Multiple regression 2	
	*p*	OR^1^ (95% CI^2^)	*p*	OR^1^ (95% CI^2^)	*p*	OR^1^ (95% CI^2^)
Gender	0.38	1.11 (0.88–1.40)	0.35	1.12 (0.88–1.45)	-	
Age > 45	< 0.001	3.27 (2.22–4.84)	< 0.001	3.43 (2.23–5.28)	< 0.001	3.54 (2.33–5.38)
Charlson Comorbidity Index > 7	< 0.001	7.62 (2.28–25.44)	0.002	6.18 (1.97–23.60)	0.002	7.35 (2.14–25.20)
Warfarin	0.17	0.67 (0.38–1.18)	0.34	0.75 (0.41–1.37)	-	
Direct oral anticoagulation	0.07	1.34 (0.98–1.82)	0.064	1.39 (0.98–1.96)	-	
Antiplatelet therapy	< 0.001	2.14 (1.62–2.82	< 0.001	1.75 (1.28–2.40)	< 0.001	1.66 (1.24–2.24)
Glasgow Coma Scale < 14	< 0.001	13.56 (7.49–24.52)	< 0.001	11.53 (5.73–23.31)	< 0.001	11.47 (5.72–22.99)
Loss of consciousness	< 0.001	2.06 (1.63–2.61)	0.002	1.55 (1.17–2.05)	0.002	1.56 (1.18–2.06)
Amnesia	< 0.001	1.92 (1.50–2.45)	< 0.001	1.74 (1.30–2.32)	< 0.001	1.75 (1.31–2.33)
Vomiting	< 0.001	2.09 (1.47–2.99)	< 0.001	2.08 (1.39–3.10)	< 0.001	2.01 (1.35–2.99)
Headache	0.68	1.06 (0.81–1.40)	-		-	
Seizure	< 0.001	4.82 (2.81–8.29)	0.005	2.45 (1.31–4.57)	0.005	2.44 (1.31–4.55)
Intoxication	0.42	0.82 (0.66–1.19)	-		-	
Scalp hematoma	0.005	1.53 (1.14–2.07)	0.032	1.42 (1.03–1.94)	0.028	1.42 (1.04–1.95)
Signs of open/depressed fracture	0.26	0.66 (0.32–1.36)	0.65	0.85 (0.41–1.76)		
Signs of skull base fracture	< 0.001	3.19 (2.07–4.90)	< 0.001	2.74 (1.74–4.31)	< 0.001	2.77 (1.76–4.37)
New neurological deficits	< 0.001	3.06 (2.15–4.35)	< 0.001	2.09 (1.39–3.13)	< 0.001	2.10 (1.40–3.15)

Table shows regression analysis in three steps. “Univariate regression” is univariate logistic regression of all relevant parameters. Multiple regression 1 is multiple logistic regression with all parameters from univariate regression that had *p* < 0.4. Multiple regression 2 is multiple logistic regression with all parameters from multiple regression 1 that had *p* < 0.05. Neither warfarin nor direct oral anticoagulation therapy is associated with intracranial hemorrhage, but antiplatelet therapy is. *R*^2^ = 0.13

^1^Odds ratio

^2^Confidence interval

## Discussion

The current study elaborates on the risk of TICH in TBI patients. It comprises a large cohort of 4850 patients with 975 patients on ACT and 673 patients on APT. The results showed that the RR for TICH in patients with APT was higher than in patients on ACT and only APT was associated with TICH in regression analysis.

Even though the risk ratio for TICH in patients on APT compared to patients on ACT was higher, this higher risk was opposed by the slight overlap of CIs. To investigate if there was an actual association between APT, ACT, and TICH, a comparison of patient characteristics and a regression analysis was executed. It could be the case that treatment with ACT or APT was simply a confounder that covaried with other associated factors of TICH such as high age or comorbidity. Nonetheless, there were no statistically significant differences between patients on ACT and APT or no medication affecting coagulation except for age and vomiting. This strengthens the findings of a higher RR of TICH in patients on APT. Furthermore, the regression analysis revealed that treatment with APT had elevated odds for TICH whereas ACT did not. This association between APT and TICH was independent of other important factors, signs, and symptoms such as age, loss of consciousness, and CCI. Therefore, it is likely that there is an actual difference in risk of TICH in head trauma patients with APT compared to those with ACT. However, this elevated risk of TICH in patients on APT is not considered in most current guidelines. This entails that TICHs could be missed in patients treated with APT. Thus, APT merits equal or more emphasis in guideline-based risk stratification of head trauma patients compared to patients on ACT [14–16, 28].

Two previous large studies, including over 55,000 patients, found that the incidence of TICH was increased in patients taking aspirin-clopidogrel, but not in patients only taking ASA [19, 33]. It is conceivable that certain subclasses of APT confer a higher risk of TICH and that guidelines can be further refined by recommending different management for different types of APT. However, no such analysis was attempted in the present study because of the distribution of different APTs with very few patients taking dual antiplatelet therapy. Furthermore, up to 30% of patients on APT do not have the intended effect of the medication and the compliance of APT 1 year after coronary intervention has been reported below 50% [40, 41]. This affects the results of the present study, but the study design makes it is difficult to assess the extent of this effect.

This study found no significant association between ACT and TICH. Thus, current recommendations to perform a head CT on all patients with ACT might not be necessary [14–16, 28]. It is conceivable that this practice leads to unnecessary CTs with increased costs, emergency department overcrowding, and increased exposure to harmful radiation. However, because of the limitations of the present study, these findings need to be corroborated in future studies. A higher TICH incidence for VKA compared to DOAC has been shown previously [20, 42]. An attempt was made in the present study to differentiate VKA from DOAC by performing a second logistic regression analyzing them separately. However, the outcome was similar to the first regression and neither VKA nor DOAC were significantly associated with TICH. Wu et al. (2022) have shown that the incidence of intracranial hemorrhage in patients on DOAC is higher in patients on rivaroxaban than apixaban and dabigatran [42]. As concluded by Fuller et al. (2020) in a recent meta-analysis, it is plausible that certain subgroups of patients within the ACT group might have a lower risk of TICH, making discretionary head CTs a feasible alternative [43]. No subgroup analysis was performed in the present study because most patients were taking apixaban and it was not deemed useful. The results of the present study that indicate a low rate of TICH in patients taking VKA affects the association between oral anticoagulation and TICH. Previous publications have shown opposite data with higher TICH rates in patients on VKA [44]. The reasons for these results cannot be determined by the present study, but it poses a limitation when interpreting the results. Prospective studies dealing with these matters in the trauma setting are warranted.

The SNC guideline used at our institution recommends a CT scan for all patients with ACT and head trauma, irrespective of severity. Primary and home care providers as well as patients are aware of this practice. It could result in more ED visits and CTs being performed after less severe traumas in the ACT cohort. This might in part explain why the present study has included more head trauma patients on ACT than on APT when prescription rates in Sweden show converse numbers. This can dilute the impact ACT has on the risk of TICH after head trauma and, at least to some degree, explain why ACT was not associated with TICH in regression analysis.

High age is considered to increase risk of TICH in present guidelines [14–16, 28]. However, the cutoff is usually at 60–65 years of age. The cutoff in the present study at 45 years might indicate that patients may need to be managed differently according to age than current guidelines prescribe. It is possible that TICHs are missed in patients younger than 60–65 years but older than 45. It is also feasible that patients under 45 years do not have the same high risk of TICH, even if they exhibit other signs and symptoms classically associated with TICH and that some CTs in this cohort are unnecessary. The exact age cutoff and what signs and symptoms that are important in the different age groups are beyond the scope of the present study. However, this finding merits further studies. Currently, we are not aware of any studies investigating this in the adult population.

A CCI more than 7 was strongly associated with TICH in the present study, but the number of patients with such a high CCI was few. Although some studies have previously identified a similar correlation between high comorbidity and TICH, current guidelines have not incorporated this in their decision algorithm [14–16, 28, 45, 46]. However, the epidemiological shift with more old patients sustaining head traumas probably entails a higher degree of comorbidity in the population. It is conceivable that certain comorbidities entail a higher risk of TICH and that the total burden of comorbidities weighted according to the CCI is not the best way to risk stratify head trauma. A study of a modified comorbidity classification system in the context of head trauma that investigates how comorbidities should be weighted might have a higher predictive value. CCI was originally intended for automatic calculation of entire cohorts based on international classification of disease codes and not individual patients which can make it time consuming to calculate manually [47].

The rest of the variables that remained in the third regression analysis are commonly associated with TICH; most of them are included in the decision algorithms of the current guidelines [14–16, 28].

Even though the sample size of the present study is relatively large, the determination coefficient of the regression analysis was only 13%. This means that a large proportion of the TICHs could not be explained by the factors included in the regression analysis. It might in part be attributed to limitations of the retrospective method and is not a measurement of the potential diagnostic accuracy of a guideline based on the current analysis. However, it is worth commenting as it indicates that TICH is very multifactorial, difficult to study and might, at least in part, explain the low specificity of the current guidelines.

The retrospective method of the present study is the biggest limitation. It can give rise to bias when interpreting medical records and dealing with missing data. Notwithstanding the measures we took to counteract this, it precludes anything but careful conclusions from this study. Our countermeasures seem to have some effect, as made evident in the Cohen’s kappa coefficient analysis. The concordance between two different data collectors was acceptable, indicating reproducibility and a certain measurement of data validity. Hence, we assume that this limitation does not affect the study’s overall conclusion.

Our geographic region adheres to the SNC guideline, which advocates for CT scans in all traumatic brain injury patients on ACT and those aged over 65 on APT. This might lead to that more patients with ACT are being sent to the hospital for scanning by primary care physicians and this might dilute and partly explain the low prevalence of ICH in the APT cohort of the present study. Furthermore, even though a head CT is recommended for patients on ACT and only recommended for patients > 65 years on APT, we still observed a higher rate of TICH in the latter group. Because of this, the true prevalence of TICH in the APT cohort might be even higher than our results indicate.

The reason for including patients by the triage code (“head injury”) was to make the analyzed cohort as representative as possible of ED patients with head injury that are managed according to head injury guidelines. This affects the constitution of the cohort and some of the most serious head injuries may not be included, but we do not believe this affects the outcomes of the present study to such an extent that that the conclusions are changed.

Trauma severity has not been quantified in the present study. It is a factor that might affect the outcome of the regression analysis, but we do not believe that it varies significantly across subgroups (e.g., ACT and APT) and because of this, its potential effect is considered small.

Most patients in the present study had mild head injury with GCS 14–15. This makes the results of the present study difficult to apply to head trauma populations with more severe head injuries.

Interpretation of “Head CT not performed” as “No TICH” might lead to overlooked intracranial hemorrhages. Nonetheless, TICHs with grave consequences were probably not missed because we screened for new ED visits 6 months after the index TBI occurred. It is possible such events might have been overlooked if they were managed outside the region Skåne, but this risk was considered minimal, and if any cases did exist, it should not vary across subgroups (e.g., ACT and APT).

## Conclusion

This study shows that antiplatelet therapy is associated with a higher risk of traumatic intracranial hemorrhage compared to oral anticoagulation. Antiplatelet therapy should be given equal or greater consideration in the guidelines compared to anticoagulation therapy. Further studies on antiplatelet subtypes within the context of head trauma are recommended to improve the guidelines’ diagnostic accuracy.

## Data Availability

Data will be made available upon request.
